# Rapid and Efficient Estimation of Pea Resistance to the Soil-Borne Pathogen *Fusarium oxysporum* by Infrared Imaging

**DOI:** 10.3390/s150203988

**Published:** 2015-02-09

**Authors:** Nicolas Rispail, Diego Rubiales

**Affiliations:** Institute for Sustainable Agriculture, CSIC, Alameda del Obispo s/n, Apdo. 4084, Córdoba 14080, Spain; E-Mail: diego.rubiales@ias.csic.es

**Keywords:** infra-red imaging system, *Fusarium oxysporum*, *Fusarium* wilt, leaf temperature, *Pisum sativum*, screening for resistance, plant breeding

## Abstract

*Fusarium* wilts are widespread diseases affecting most agricultural crops. In absence of efficient alternatives, sowing resistant cultivars is the preferred approach to control this disease. However, actual resistance sources are often overcome by new pathogenic races, forcing breeders to continuously search for novel resistance sources. Selection of resistant accessions, mainly based on the evaluation of symptoms at timely intervals, is highly time-consuming. Thus, we tested the potential of an infra-red imaging system in plant breeding to speed up this process. For this, we monitored the changes in surface leaf temperature upon infection by *F. oxysporum* f. sp. *pisi* in several pea accessions with contrasting response to *Fusarium* wilt under a controlled environment. Using a portable infra-red imaging system we detected a significant temperature increase of at least 0.5 °C after 10 days post-inoculation in the susceptible accessions, while the resistant accession temperature remained at control level. The increase in leaf temperature at 10 days post-inoculation was positively correlated with the AUDPC calculated over a 30 days period. Thus, this approach allowed the early discrimination between resistant and susceptible accessions. As such, applying infra-red imaging system in breeding for *Fusarium* wilt resistance would contribute to considerably shorten the process of selection of novel resistant sources.

## 1. Introduction

*Fusarium* wilts are widespread diseases caused by many forms of the soil-borne pathogen *Fusarium oxysporum*, affecting many agricultural crops, including most legumes, cucurbits, tomato, strawberry, cotton and banana [1,2]. This soil-borne pathogen can survive as thick-walled chlamydospores, which remain viable in the soil for many years, which makes its control difficult. Upon host recognition, the germinating fungus is able to penetrate the root and reach the vascular vessels where it grows profusely leading to a rapid plant death, in part due to drastic water stress [3]. Once established, this pathogen is very difficult to eradicate since it can grow in the absence of a compatible host.

In absence of alternative efficient or economically viable methods of control, the use of resistant cultivars is the most used approach to control *Fusarium* wilt. However sources of resistance are somewhat limited and single genes, which have been identified and used in breeding, are rapidly overcome by new races of the pathogen [4]. It is thus essential to continuously search for novel sources of resistance to complement and reinforce the actual resistance of elite cultivars. This search requires the availability of large germplasm collections and of precise and accurate screening techniques [4]. Efficient screening methods have been established and described for many crops [4–8] but these are highly time-consuming. Indeed, symptoms usually initiate on oldest leaves around 7–10 days post-inoculation (dpi) following artificial inoculation in growth room and they will progress through the plant until complete plant death that usually occurs around 20 to 30 dpi in susceptible genotypes. Late wilting or moderately resistant genotypes may die even later. Thus the whole process requires more than one month to be completed under controlled environments. In field conditions with natural inoculation an accurate evaluation would require much more time. Alternative disease evaluation methods accelerating the screening and selection of resistant lines would thus be advantageous.

The recent development of imaging-based phenotyping such as the evaluation of surface temperature by infra-red imaging system and measurement of chlorophyll fluorescence is revolutionizing agriculture and plant science. Infra-red thermal imaging is a non-contact, non-destructive and rapid technique which provides a temperature map of the targeted material or plant. In addition, the infra-red camera is easy to handle and highly accurate making it suitable for both laboratory and field studies [9]. In general, plant surface temperature is dependent on transpiration rate. At high transpiration rate, the leaf temperature is cooling while at lower transpiration rate, this temperature increases. Surface leaf temperature is thus an indirect parameter to evaluate the overall physiological status of a plant [10]. As such, it has been largely applied to monitor plant water status [11]. It is also powerful to identify stressed plants by both biotic and abiotic stresses [12]. Thus, this method has been applied to detect the presence of several plant diseases both in field and laboratory indicating that infra-red imaging could be used to detect vascular wilt diseased plants [10,13–16]. Application of thermal imaging or thermography approaches offers great potential for breeding [17]. For instance, this approach was successfully used to screen for barley and Arabidopsis mutants deficient in stomatal movements [18,19] and to identify drought or salinity tolerant genotypes in rice [20], barley [21], wheat [22] and maize [23]. However, the potential of these methods in breeding for disease resistance has not been explored yet. Thus, we used the pea-*F. oxysporum* pathosystem as a model to evaluate the potential of infra-red thermal imaging as an indirect screening method to discriminate susceptible and resistant plants speeding up the evaluation of *Fusarium* wilt resistance in crops and the selection of resistant accessions.

## 2. Results

**Figure 1.**
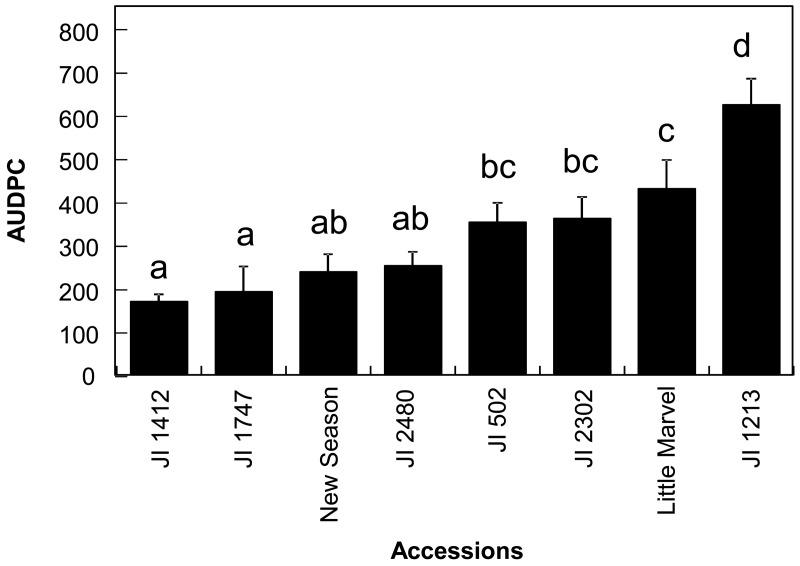
Assessment of *Fusarium oxysporum* f. sp. *pisi* (*Fop*) disease symptoms in the selected *Pisum sativum* accessions. The histogram shows the area under the disease progression curve (AUDPC) calculated for each *P. sativum* accession from the periodic evaluation of *Fop* disease symptoms expressed as percentage of wilting leaves per plant. Vertical bars are standard errors for n = 10. Different letters between each histogram indicates significant difference between values according to Duncan multiple range test at α = 0.05.

Prior to the infra-red analysis of the accessions, we evaluated their responses to the disease from 7 to 30 dpi in order to estimate the area under the disease progression curve (AUDPC) for further analysis and to confirm their resistance status. As expected, we detected continuity in the disease response of the different accessions from resistant to susceptible (Figure 1). The disease response of the accessions was broadly similar to that previously reported [5]. Significant differences were detected between accessions (*p* < 0.001) and mean comparison by Duncan multiple range test allowed the discrimination between the resistant accessions JI 1412, JI 1747, JI 2480 and New Season from the moderately susceptible accessions JI 502, JI 2302 and Little Marvel while the last accession JI 1213 was classified as susceptible (Figure 1).

**Figure 2.**
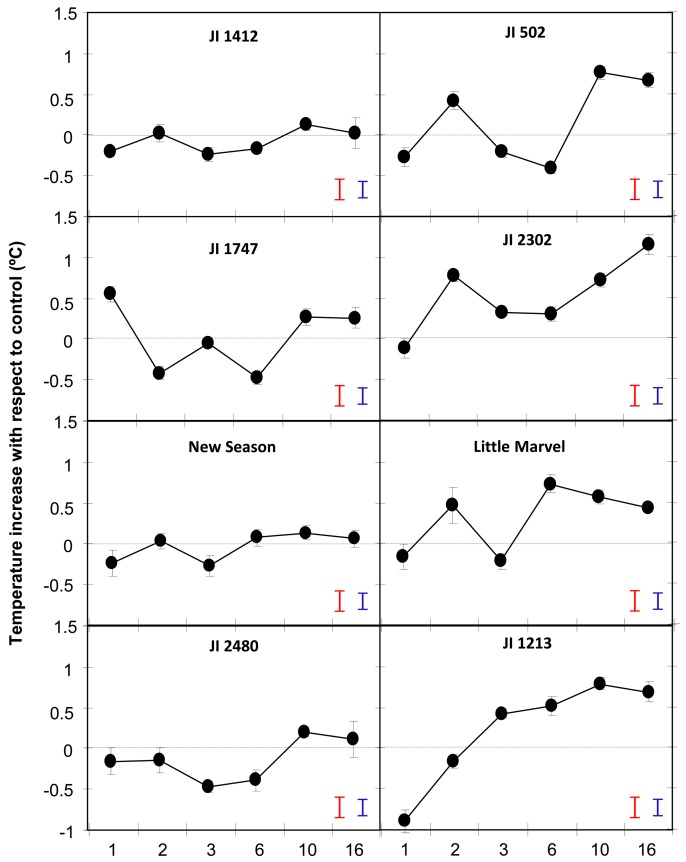
Evolution of the surface leaf temperature of *P. sativum* plants following inoculation with *F. oxysporum* f. sp. *pisi* (*Fop*). The graphs represent the evolution of the differences in surface temperature between *Fop*-inoculated and control plants for the resistant accessions JI 1412, JI 1747, JI 2480 and New Season, the partially resistant, JI 502, JI 2306 and Little Marvel, and the susceptible accession JI 1213 according to the time post-inoculation. Surface leaf temperatures were measured by infra-red imaging system. Vertical bars on each data point are standard errors for n = 10. Red and blue vertical bars represent the LSD values at α = 0.05 between genotypes (LSD = 0.26) and time points (LSD = 0.21), respectively.

Comparison of the surface leaf temperature between control and inoculated plants showed an early and slight reduction of temperature in all accessions following inoculation (Figure 2). Thereafter, temperature oscillated in most accessions during the first 6 dpi, in most cases following a similar trend and probably reflecting plant adaptation to the uneven stresses applied at inoculation time. After that initial period, the leaf temperature of inoculated resistant accessions stabilized to control level while that of moderately and highly susceptible accessions increased more than 0.5 °C (Figure 2).

**Figure 3.**
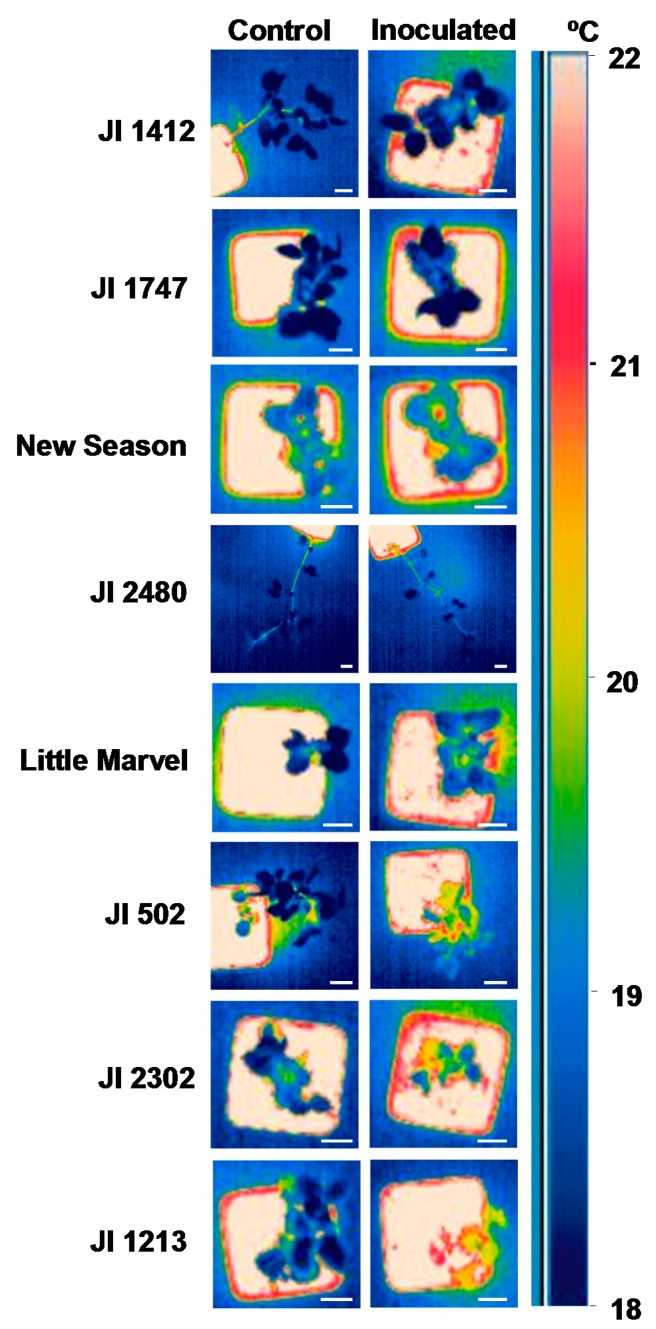
Comparison of the surface leaf temperature between control and inoculated *P. sativum* plants with *F. oxysporum* f. sp. *pisi (Fop)* at 10 days post-inoculation. The figure shows a representative image of each accession maintained non-inoculated (Control, **Left**) and inoculated with *Fop* (**Right**). Scale bars indicate 2 cm.

Thus by 10 dpi the temperature of JI 1412, JI 1747, JI 2480 and New Season was not different from that of control, non-inoculated, plants (Figure 2). This was also detectable by visual observation of the snapshots obtained with the camera (Figure 3). By contrast moderately and highly susceptible plants increased its leaf temperature between 0.6 and 0.85 °C in respect to their non-inoculated controls (Figure 2). This slight increase of temperature was also appreciated visually in the images (Figure 3).

**Figure 4.**
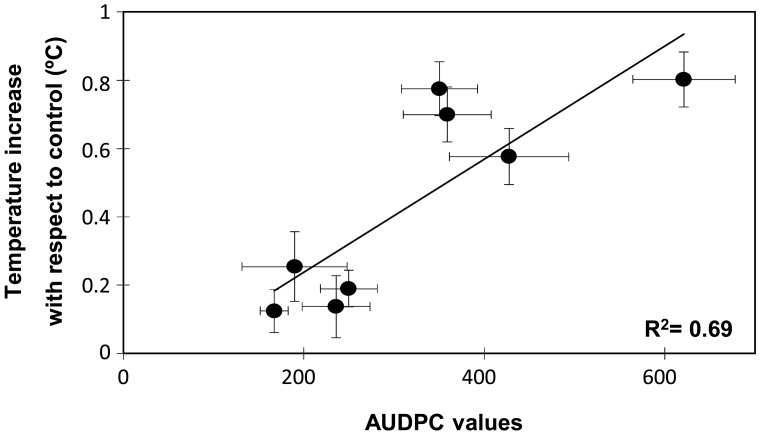
Relationship between the level of susceptibility to *F. oxysporum* f. sp. *pisi* (*Fop*) and surface leaf temperature. The graphic represents the linear correlation calculated for the differences in surface leaf temperature detected for each *P. sativum* accession between inoculated and control plants at 10 days post-inoculation with *Fop* in relation with their corresponding AUDPC values. Horizontal bars are standard errors for AUDPC values with n = 10 while vertical bars are standard errors for the differences in surface leaf temperature calculated with n = 10.

In order to determine whether the temperature increases and the susceptible/resistance responses were statistically correlated, a Spearman rank correlation analysis was performed (Table 1). As expected the disease rating at 10 or 16 dpi and the AUDPC values were highly correlated among them. Interestingly, Spearman rank correlation also indicated a positive and significant correlation between temperature increases at 10 dpi and the disease symptoms either recorded as disease ratings at 10 (0.551, *p* < 0.001) or 16 dpi (0.628, *p* < 0.001) or as the AUDPC value (0.658, *p* < 0.001). A significant correlation was also detected between temperature increases at 16 dpi and disease ratings or AUDPC. However at this time the correlation coefficient was smaller with a mean of 0.396. Further, when plotting the changes in leaf temperature at 10 dpi against the AUDPC values two groups were also clearly discriminated, one of them comprising the resistant accessions and the other clustering the moderately and highly susceptible accessions (Figure 4). The regression analysis showed that data fitted (R^2^ = 0.694) a linear regression curve with a mean difference of temperature of 0.5 °C between the resistant and susceptible genotype groups.

## 3. Discussion

Infra-red thermography is a very powerful method to monitor changes in plant water status *in vivo*. Thus it is being largely used in agriculture and plant science for a wide range of applications, from scheduling irrigation to evaluating fruit maturity [9,24]. However, the usefulness of thermal imaging has not been sufficiently explored in disease resistance breeding. Here, we show that infra-red thermography allows the detection of pea plants infected by the vascular wilt pathogen *F. oxysporum* and the discrimination between susceptible and resistant pea cultivars at a very early stage of the interaction. As such infra-red thermography might be very useful for future screening for vascular wilt resistance in germplasm collections of pea and other plant species.

Surface leaf temperature is strongly correlated with the plant transpiration rate. Thus, during water stress-induced stomata closure the transpiration rate decreased and as a result, the surface leaf temperature increased compared to well-watered control plants [11,25]. A rapid closure of stomata has also been detected in response to infection by air-borne pathogens leading to an increase in surface leaf temperature [10,26]. This has led to the application of remote sensing approaches to detect foliar diseases in the field [13,15]. Interestingly, in plants infected by fungal root pathogens, such as *F. solani*, *Rhizoctonia solani*, *Verticillium dahliae,* a higher surface temperature with respect to healthy plants has been observed [13,27]. Changes in stomatal conductance, transpiration rate and superficial leaf temperature in susceptible wilted plants upon infection by *F. oxysporum* have also been reported [16,28,29]. Accordingly, we detected an increase in the leaf temperature of susceptible accessions of pea upon infection with the soil-borne pathogen *F. oxysporum* (Figures 2 and 3). This reinforced the close relationship previously established between vascular wilt and drought [28,30].

From a mechanistic point of view several processes might account for the temperature increase in the susceptible accessions. It is often believed that the wilting and death of *F. oxysporum*-infected plants are due to perturbation of the water fluxes within the plants [28,30]. The increase in leaf temperature and associated stomatal closure might be related to the vessel plugging induced by the intensive fungal growth within xylem vessels and by the attempt of plant defence that blocked xylem cells [16,30]. However, recent studies showed that the water flux while disturbed was not blocked by these defence responses [28]. In accordance with this, we did not observe significant changes of the surface leaf temperature in the resistant pea accessions (JI 1412, JI 1747, JI 2480 and New Season) upon infection. Alternatively the temperature increase observed in the susceptible plants might be related to toxin production [28,29,31]. Fusaric acid has been shown to be one of the main toxins produced by *F. oxysporum* [32] and an important pathogenicity factor since it induces wilting symptoms in several plant species, including cucumber, banana and pepper [28,29,31–33]. Several studies in banana, cucumber and watermelon showed that application of fusaric acid to the root led to a rapid ABA-dependent stomatal closure. As such, the application of fusaric acid mimicked the temperature rise of the leaf detected upon infection with the pathogen in these species [28,29,31]. Independently of the causal effect leading to stomatal closure and transpiration rates, this temperature increase was only detected in susceptible accessions since the surface leaf temperature of resistant pea accessions was maintained at control level (Figure 2). Thus by monitoring the changes in leaf temperature, we were also able to differentiate the response observed between the resistance and susceptible accessions at an early stage of the interaction which opens the possibility to use infra-red thermography in *Fusarium* wilt resistance breeding.

The genotype-dependent oscillations of temperature observed in most accessions during the first 6 dpi probably reflected the plant adaptation to the inoculation process and the attempt of plant defence. Thereafter, the surface leaf temperature rise of about 0.5 °C for all susceptible accessions while it remained at control level in the resistant accessions (Figure 2). In addition, the changes in leaf temperature measured at either 10 or 16 dpi were positively correlated with the disease symptom rates measured at 16 dpi and by the AUDPC (Table 1 and Figure 4). Surprisingly, the correlation between the disease parameters and the leaf temperature changes was higher when the surface temperature was measured at 10 dpi than at 16 dpi. Although disease symptoms were only initiating at 10 dpi, a previous study showed that the fungus had already colonized the whole plant of susceptible pea accession at this stage [5]. Thus at this stage, the temperature difference might reflect the pathogen action within susceptible plants. By contrast, in the resistant accessions, the pathogen only colonized the root system being efficiently blocked at crown level by the establishment of defence reactions [5]. This might explain that the leaf temperature of inoculated resistant accessions rally control level by 10 dpi (Figure 2). At days 16, disease symptoms are clearly visible on all susceptible plants and on some older leaves of some resistant accessions. The presence of dying leaves on these plants may distort the leaf temperature of the plant which might explain the lower correlation observed between disease symptom and temperature at this later stage. Altogether this suggests that the measurement of leaf temperature at 10 dpi better reflect the plant physiological response to the pathogen.

Although the changes in the temperature between accessions following pathogen attack were relatively small, it allowed separating the accessions in two groups according to their level of susceptibility before the appearance of the initial visual symptoms (Figures 2 and 3) and hence, it might be very valuable to identify and select resistant accessions. From a breeding perspective, while several methods of inoculation have been developed to allow rapid and reproducible *Fusarium* wilt [5,34], the identification of resistant accessions to this disease is mainly based on the evaluation of symptoms at timely intervals which is time-consuming [4,5]. The application of thermal imaging system to monitor changes in leaf temperature at an early stage of the interaction may contribute to reduce the time needed for the identification and selection of resistant pea plants to *Fusarium* wilt.

Screenings for resistance to soil-borne diseases are performed either under field condition or under a controlled environment. While field testing is often considered a more appropriate approach, the uneven distribution of pathogenic population in the soil and the presence of additional soil pathogen may interfere with the results [4]. To circumvent these drawbacks, screening approaches under controlled environments have been developed and shown to be well-correlated with field testing. Although, the limited space is often an important issue, this approach allows for large-scale screening of germplasm collections [4,5,8]. Surface temperature is strongly affected by environmental conditions thus this method may not be adequate for field screening which are subject to very variable environmental conditions. However, implementation of infra-red thermography may be very useful to speed up the screening of large germplasm collections under controlled environments in which plants are maintained in more stable and constant conditions. This is supported by the fact that resistant accessions were clearly separated from the susceptible ones when comparing the image thermography of control and inoculated plants at 10 and to a lesser extent at 16 dpi (Figure 3). The significant and positive correlation between the AUDPC values and the changes in leaf temperature at 10 dpi also support that leaf surface temperature at this time can be used as indirect parameter for selection under controlled environment. Although detailed evaluation of disease symptoms might be more accurate, it is highly time-consuming. Detailed analyses of infra-red thermography images and calculation of the overall surface temperature of all plants is also time-consuming. However, visual monitoring of the infra-red thermography images was found sufficient to discriminate between resistant and susceptible accessions as shown in Figure 3 and is a very rapid approach since it can be done during image acquisition without further image processing. As a result, the application of infra-red thermography may reduce the time needed to screen large germplasm collections.

Altogether, our results support the usefulness of thermal imaging system to screen large pea germplasm collections for *F. oxysporum* disease resistance or to select resistant individuals within large segregating populations under controlled environment. Changes in temperature or transpiration rate have been detected in susceptible accessions of many plant species in response not only to *Fusarium* wilt disease but also many other air-borne and soil-borne diseases [16,28,34–36]. Such changes in stomatal conductance or transpiration rate have not been detected or only transiently in resistant accessions (Figure 2) [36,37] suggesting that this can be an interesting parameter for screening for resistance not only for *Fusarium* wilt but also to other important diseases such as powdery mildew, rust or root rots.

## 4. Experimental Section

### 4.1. Fungal Isolate and Culture Conditions

The *Fusarium oxysporum* f. sp. *pisi* (*Fop*) race 2 strain R2F42 was used in all the experiments. The fungal strain was stored as microconidial suspensions at −80 °C in 30% glycerol. For microconidia production, cultures were grown in potato dextrose broth (PDB; Difco, Detroit, MI, USA) at 28 °C in a shake culture set at 170 rpm.

### 4.2. Plant Material and Growth Conditions

Eight accessions of *P. sativum* (JI 502, JI 1213, JI 1412, JI 1747, JI 2302, JI 2480, Little Marvel and New season) with contrasting responses to *Fop* were used in this study [5]. Pea seeds were surface-sterilized for 20 min in a 20% solution of sodium hypochlorite and then rinsed three times with sterile water before being wrapped in wet filter paper in a Petri dish. Then the seeds were stratified for two days at 4 °C in the dark and incubated at 20 ± 2 °C until germination. Once germinated, the seedlings were transferred to pots (36 cm^2^ × 8 cm) containing sterile vermiculite (2–8 mm diameter) and grown in a controlled environmental chamber under a 16/8 h light-dark photoperiod at 26 ± 2 °C temperature regime with 200 μmol·m^−2^·s^−1^ of illumination. Plants were watered every three days with tap water.

### 4.3. Inoculation and Disease Assessment

Seven-day-old pea seedlings (2–3 node stage) were inoculated following the root dipping method previously described [5]. For this, each plant were uprooted, cleaned off vermiculite and the whole root system was submerged for 30 min in a solution of 5 × 10^6^ *Fop* microconidia·mL^−1^ (inoculated plants) or in sterile water (control plants). Seedlings were then planted in individual pots containing sterile vermiculite and placed back in the growth chamber with the same growth condition as indicated above. 10 plants per accession were used for each treatment and organized in two blocks of 5 plants per accession. In each block, inoculated plants were maintained in a tray near the control plants. In addition, blocks were shuffled around the growth chamber every three days to homogenize the growth condition between blocks. *Fop* symptoms were rated every three days from 7th to 30th dpi by estimating the percentage of leaves with symptoms per plant. At the end of the experiment, these data were used to calculate the AUDPC value as described previously [5].

### 4.4. Thermal Imaging of Control and Inoculated Plants

Infra-red images of pea plants were obtained with a Thermovision A40M (FLIR, Wilsonville, OR, USA) thermal camera equipped with a 43° FOV lens and connected to computer via the IEEE-1394 protocol. The image sensor was a Focal Plane Array (FPA) based on uncooled microbolometers with a resolution of 320 × 240 pixels, a spectral response in the range 7.5–13 μm, with 0.08 °C sensitivity at 30 °C and 0.1 mm minimal focus distance. Digital thermograms were acquired with the temperature range set between +10 and +55 °C with the spectral rainbow color scheme and the autoadjust function in off with FSCAP software (FLIR). The leaf temperature for each plant was determined by calculating the mean temperature of four spots (3 mm diameter) placed on four distinct leaves. Leaf temperature was monitored at 1, 2, 3, 6, 10 and 16 dpi.

### 4.5. Statistical Analysis

Differences in surface leaf temperature and in disease severity between accessions were statistically assessed by ANOVA using contrast analysis (Scheffe) and Duncan multiple range test respectively. Spearman rank correlation test were performed to determine correlations between changes in superficial leaf temperature. All statistical analyses were performed with SPSS Statistics v. 22 software (IBM Corp., Armonk, NY, USA).

## 5. Conclusions

In this study, the possibility to use infra-red thermography for disease resistance breeding was evaluated. Using the pea-*F. oxysporum* pathosystem, we demonstrated that infra-red thermography can not only indicate the presence of this soil-borne pathogen within susceptible pea plants but also discriminate between resistant and susceptible plants at an early stage of the interaction under controlled environment. In this pathosystem, a significant increase of the superficial leaf temperature was detected as early as 10 dpi for the susceptible accessions while the temperature of resistant accessions rallied control level. The temperature increase in susceptible accessions was positively correlated with the disease symptoms estimated over a period of 30 days after inoculation. Thus the implementation of infra-red thermography in breeding for resistance to *Fusarium* wilt may reduce the time required to identify and select resistant plants while screening large germplasm pea collections under controlled environment. Since infection by other plant pathogens also led to an increase of temperature in susceptible plants from different species, the usefulness of infra-red thermography in resistance breeding may be expanded to additional diseases and crops.

## Figures and Tables

**Table 1.** Spearman rank correlation coefficient for disease symptom levels and changes in surface leaf temperature comparisons for n = 10.

	**Disease Rating 10 dpi**	**Disease Rating 16 dpi**	**AUDPC Value**	**T**° **Increase at 10 dpi**	**T**° **Increase at 16 dpi**
Disease rating 10 dpi	1.000	-	-	-	-
Disease rating 16 dpi	0.631 ***	1.000	-	-	-
AUDPC value	0.902 ***	0.891 ***	1.000	-	-
T° increase 10 dpi	0.540 ***	0.628 ***	0.658 ***	1.000	-
T° increase 16 dpi	0.321 **	0.442 ***	0.424 ***	0.380 **	1.000

** and

*** indicates the significance of the correlation coefficient according to the Student *t* test at 0.01 and 0.001 level respectively.
